# Snail/PRMT5/NuRD complex contributes to DNA hypermethylation in cervical cancer by TET1 inhibition

**DOI:** 10.1038/s41418-021-00786-z

**Published:** 2021-05-05

**Authors:** Jie Gao, Ruiqiong Liu, Dandan Feng, Wei Huang, Miaomiao Huo, Jingyao Zhang, Shuai Leng, Yang Yang, Tianshu Yang, Xin Yin, Xu Teng, Hefen Yu, Baowen Yuan, Yan Wang

**Affiliations:** 1 Tianjin Key Laboratory of Inflammatory Biology, The province and ministry co-sponsored collaborative innovation center for medical epigenetics, Department of Biochemistry and Molecular Biology, School of Basic Medical Sciences, Tianjin Medical University, Tianjin, China; 2 The Second Hospital, Cheeloo College of Medicine, Shandong University, Jinan, Shandong China; 3 Beijing Key Laboratory of Cancer Invasion and Metastasis Research, Advanced Innovation Center for Human Brain Protection, Department of Biochemistry and Molecular Biology, School of Basic Medical Sciences, Capital Medical University, Beijing, China; 4 Key Laboratory of Cancer and Microbiome, State Key Laboratory of Molecular Oncology, National Cancer Center/National Clinical Research Center for Cancer/Cancer Hospital, Chinese Academy of Medical Sciences and Peking Union Medical College, Beijing, China

**Keywords:** Metastasis, Epigenetics

## Abstract

The biological function of PRMT5 remains poorly understood in cervical cancer metastasis. Here, we report that PRMT5 physically associates with the transcription factor Snail and the NuRD(MTA1) complex to form a transcriptional-repressive complex that catalyzes the symmetrical histone dimethylation and deacetylation. This study shows that the Snail/PRMT5/NuRD(MTA1) complex targets genes, such as *TET1* and *E-cadherin*, which are critical for epithelial-mesenchymal transition (EMT). This complex also affects the conversion of 5mC to 5hmC. This study demonstrates that the Snail/PRMT5/NuRD(MTA1) complex promotes the invasion and metastasis of cervical cancer in vitro and in vivo. This study also shows that PRMT5 expression is upregulated in cervical cancer and various human cancers, and the PRMT5 inhibitor EPZ015666 suppresses EMT and the invasion potential of cervical cancer cells by disinhibiting the expression of TET1 and increasing 5hmC, suggesting that PRMT5 is a potential target for cancer therapy.

## Introduction

Protein arginine methyltransferase 5 (PRMT5) is a type II protein arginine methyltransferase (PRMT) that has been reported to catalyze the symmetrical dimethylation of arginine [1]. PRMT5 is linked to gene silencing through repressive histone markers, including symmetrical dimethylation of H4R3 and H3R8 [2–4]. MEP50 is a cofactor, which directly binds to PRMT5 and increases its affinity for substrates [5–7]. Additionally, PRMT5 can participate in multiple cellular processes, including cell proliferation, DNA replication, cell cycle, and cell invasion and metastasis by binding to a variety of epigenetic regulators and regulating the expression of target genes [8–11]. Genetic depletion of PRMT5 has been reported to impair the viability of cancer cells by promoting G1 cell cycle arrest and apoptosis [12]. The transcriptional-repressive function of PRMT5 is also crucial for epithelial-mesenchymal transition (EMT), a hallmark of tumor progression [13, 14]. PRMT5 is also involved in regulating multiple signaling pathways [15, 16]. PRMT5 inhibitors have recently emerged in clinical trials for multiple solid and blood malignancies [17, 18]. However, the molecular mechanism and function of PRMT5 in the metastasis of cervical cancer requires further analysis.

The transcription factor Snail belongs to the C2H2 superfamily and is an important regulator of cell migration in embryonic development and cancer metastasis by activating the EMT program through direct inhibition of E-cadherin transcription [19]. Snail contains C-terminal tandem zinc finger motifs and an N-terminal SNAG repression domain [20, 21]. The zinc finger motifs of Snail can recognize the E-box sequence of target genes and the SNAG domain can recruit a variety of repressive cofactors. Snail can recruit the polycomb repressive complex 2 (PRC2), G9A, SUV39H1, corepressor SIN3A, HDAC1, 2 and 3, and LSD1, which coordinate histone modifications such as methylation and acetylation of H3K4, H3K9, and H3K27 [22–25]. These studies demonstrated that Snail plays an important role in tumor metastasis and recurrence.

The Mi-2/nucleosomal remodeling and deacetylase (NuRD) complex is one of the ATP-dependent chromatin remodeling protein complexes and is highly conserved among eukaryotes [26, 27]. The NuRD complex is an important epigenetic regulator of chromatin structure and transcriptional repression [28, 29]. It has been reported that PRMT5 associates with and symmetrically dimethylates MBD2, which is an important component of NuRD complex [30]. The metastasis tumor antigen (MTA) family proteins MTA1, MTA2, and MTA3 are integral components of the Mi-2/NuRD complex and act as transcription regulators [31, 32]. It is thought that different MTAs generally exist in exclusive NuRD complexes and do not co-localize in the same complex [33]. Although all MTAs have been implicated in cancer progression and metastasis, MTA1 and MTA3 play opposing roles in the EMT process [29, 34–36]. The expression of MTA1 is correlated with the aggressive ability of many cancers, indicating that MTA1 is a potential cancer therapeutic target [36].

In this study, we found that PRMT5 coordinates with Snail and the NuRD(MTA1) complex to achieve transcriptional silencing of target genes including *TET1*, *E-cadherin*, and others. We demonstrated that PRMT5 promotes the invasion and tumorigenesis of cervical cancer and its expression is markedly upregulated in various human cancers. Our data suggest that the PRMT5 inhibitor EPZ015666 can lead to an induction of *TET1* expression and 5hmC, and inhibit the invasive potential of cervical cancer cells, making PRMT5 a potential target for cancer diagnosis and treatment.

## Materials and methods

### Antibodies and reagents

The following antibodies were used: anti-PRMT5 (ab109451), anti-H3 (ab32107), anti-MTA1 (ab71153), anti-MTA2 (ab50209), anti-H4R3me2s (ab5823), anti-H4R3me2a (ab129231), anti-H3R8me2s (ab130740), anti-H3R8me2a (ab127163), anti-5-hmc (ab106918), and anti-5-mc (ab10805) (Abcam, Cambridge, UK); anti-WDR77/MEP50 (A301-561A) (Bethyl Laboratories, Montgomery, TX, USA); anti-HDAC1 (H3284), anti-HDAC2 (H3159), anti-RbAp46/48 (R3779), anti-b-actin (A1978), anti-Fibronectin (F3648), anti-Vimentin (V6630), anti-GAPDH (G8795), anti-FLAG (F3165), and anti-HA (H6908) (Sigma–Aldrich, St. Louis, MO, USA); anti-Mi-2 (SC11378), anti-MBD3 (SC271521), anti-OGT (SC32921); anti-DDB1 (SC25367), and normal IgG R/M (Santa Cruz Biotechnology, Dallas, TX, USA); anti-MTA3 (IM1012) (Millipore, Billerica, MA, USA); anti-Snail (13099-1-AP) (Proteintech, Rocky Hill, NJ, USA); anti-E-cadherin (610181), anti-N-cadherin (610920), anti-α-Catenin (610193), and anti-γ-Catenin (610253) (BD Biosciences, Franklin Lakes, NJ, USA); anti-TET1 (91171) (ACTIVE MOTIF, Carlsbad, CA, USA). Protein A/G Sepharose CL-4B beads were from Amersham Biosciences (Amersham, UK), and protease inhibitor mixture cocktail was from Roche Applied Science (Basel, Switzerland). The short hairpin RNAs (shRNAs) were obtained from GenePharma Co., Ltd. (Shanghai, China) and all short interfering RNAs were from Sigma–Aldrich. The targeted sequences are listed in Supplementary File 1 (Supplemental Table S1).

### Cell culture and transfection

The cell lines HeLa and SiHa used were obtained from the American Type Culture Collection (ATCC, Manassas, VA, USA) and maintained in Dulbecco’s modified Eagle’s medium (Biological Industries, Beit-Haemek, Israel). The Ca Ski cell line was obtained from ATCC and maintained in RPMI-1640 medium (Biological Industries). All cell culture media were supplemented with 10% fetal bovine serum, 100 U/mL penicillin, and 100 U/mL streptomycin (Gibco BRL, Grand Island, NY, USA). Cells were maintained in a humidified incubator equilibrated with 5% CO_2_ at 37 °C. All the cell lines were authenticated by STR profiling and tested for mycoplasma contamination. Transfections were carried out using Lipofectamine 2000 or Lipofectamine^®^ RNAiMAX Reagent (Invitrogen, Carlsbad, CA, USA) according to the manufacturer’s instructions. Each experiment was performed in triplicate and repeated at least three times. For RNAi experiments, at least two independent shRNA sequences were tested for each gene and that showing the highest efficiency was used. Stable cell lines expressing Snail, PRMT5, or MTA1 were generated by transfection of pCMV-Tag2B-Flag-Snail, PRMT5, or MTA1 and screened for expression in single colonies in the presence of 1 mg/mL G418. Recombinant lentiviruses expressing shPRMT5, shSnail, and shMTA1 were constructed by Shanghai GenePharma (Shanghai, China). Concentrated viruses were used to infect 5 × 10^5^ cells in a 60-mm dish with 8 μg/mL polybrene in the medium. Infected cells were then sorted for target expression.

### Immunopurification and mass spectrometry

HeLa cells stably expressing HA-PRMT5 were generated by transfecting the cells with HA-tagged PRMT5 followed by selection in medium containing 1 mg/mL of G418. Anti-HA immunoaffinity columns were prepared using anti-HA affinity gel (Sigma–Aldrich) following the manufacturer’s instructions. Cell lysate was obtained from ~5 × 10^8^ cells and applied to an equilibrated HA column with a 1 mL bed volume to allow protein complexes to bind the column resin. After binding, the column was washed with cold BC500 buffer containing 50 mM Tris, 2 mM EDTA, 500 mM KCl, 10% glycerol, and protease inhibitors. HA peptide (0.2 mg/mL, Sigma–Aldrich) was applied to the column to elute the HA protein complex as described by the manufacturer. Fractions of the bed volume were collected and resolved on sodium dodecyl sulfate (SDS)-polyacrylamide gels, silver stained, and subjected to liquid chromatography-tandem mass spectrometry (LC-MS/MS) sequencing and data analysis.

### Fast protein liquid chromatography (FPLC)

HeLa nuclear extracts were prepared and dialyzed against buffer D (20 mM HEPES, pH 8.0, 10% glycerol, 0.1 mM EDTA, 300 mM NaCl) (Applygen Technologies, Beijing, China). Approximately 6 mg of nuclear protein was concentrated to 1 mL using a Millipore Ultrafree centrifugal filter apparatus (10 kDa nominal molecular mass limit), and then applied to an 850 × 20 mm Superose 6 size exclusion column (Amersham Biosciences) that had been equilibrated with buffer D containing 1 mM dithiothreitol and calibrated with protein standards (blue dextran, 2000 kDa; thyroglobulin, 667 kDa; Ferritin, 440 kDa; Aldolase, 158 kDa; Ovalbumin, 43 kDa; all from Amersham Biosciences). The column was eluted at a flow rate of 0.5 mL/min and fractions were collected.

### Immunoprecipitation and western blotting

For immunoprecipitation assays, the cells were washed with cold phosphate-buffered saline (PBS) and lysed with cold cell lysis buffer for 30 min at 4 °C. Next, 500 μg of cellular extract was incubated with the appropriate specific antibodies or normal rabbit/mouse immunoglobin G (IgG) on a rotator at 4 °C overnight with constant rotation, followed by the addition of protein A/G Sepharose beads and incubation for 2 h at 4 °C. The beads were washed five times with cell lysis buffer (50 mM Tris-HCl, pH 7.4, 150 mM NaCl, 1 mM EDTA, 0.5% NP-40, 0.25% sodium deoxycholate, and protease inhibitor mixture). The immune complexes were subjected to SDS-polyacrylamide gel electrophoresis (PAGE) followed by immunoblotting with secondary antibodies. Immunodetection was performed using enhanced chemiluminescence (ECL System, Thermo Fisher Scientific, Waltham, MA, USA) according to the manufacturer’s instructions.

### Glutathione S-transferase (GST) pull-down experiments

GST fusion constructs were expressed in *Escherichia coli* BL21 cells, and crude bacterial lysates were prepared by sonication in cold PBS in the presence of a protease inhibitor mixture. In vitro transcription and translation experiments were performed with rabbit reticulocyte lysate (TNT Systems; Promega, Madison, WI, USA). In GST pull-down assays, approximately 10 μg of the appropriate GST fusion protein was mixed with 5–8 μL of the in vitro transcribed/translated products and incubated in binding buffer (0.8% bovine serum albumin in PBS in the presence of the protease inhibitor mixture). The binding reaction was then added to 30 μL of glutathione-Sepharose 4B beads (GE Healthcare, Little Chalfont, UK) and mixed at 4 °C for 2 h. The beads were washed five times with binding buffer, resuspended in 30 μL of 2× SDS-PAGE loading buffer, and resolved on 12% gels. Protein bands were detected with specific antibodies by western blotting.

### Chromatin immunoprecipitation (ChIP) and Re-ChIP

ChIPs and Re-ChIPs were performed in HeLa cells as previously described [28]. Briefly, 1 × 10^7^ cells were crosslinked with 1% formaldehyde, sonicated, precleared, and incubated with 5–10 μg of antibody in each reaction. Complexes were washed with low- and high-salt buffers, and DNA was extracted and precipitated. For Re-ChIP assays, immune complexes were eluted from the beads with 20 mM dithiothreitol. Eluents were then diluted by 30-fold with ChIP dilution buffer and subjected to a second immunoprecipitation reaction. The final elution step was performed using 1% SDS solution in Tris-EDTA buffer, pH 8.0. DNA template enrichment was analyzed by conventional polymerase chain reaction (PCR) using primers specific for each target gene promoter. The bands were then inverted to black by Photoshop to make it easier to recognize. The primers sequences are shown in Supplementary File 1 (Supplemental Table S2).

### ChIP sequencing

HeLa cells were maintained in DMEM supplemented with 10% fetal bovine serum. Approximately 5 × 10^7^ cells were used for each ChIP-seq assay. The chromatin DNA precipitated by polyclonal antibodies against PRMT5, MTA1 or Snail. The DNA was purified with the Qiagen PCR purification kit. In-depth whole genome DNA sequencing was performed by the CapitalBio Corporation, Beijing. The raw sequencing image data were examined by the Illumina analysis pipeline, aligned to the unmasked human reference genome (NCBI v36, hg18) using ELAND (Illumina), and further analyzed by MACS (Model-based Analysis for ChIP-Seq). Enriched binding peaks were generated after filtering through the input data. De novo motif screening was performed on sequences ±125 bp from the centers of binding peaks using the CEAS and MEME systems. Pathway analysis was conducted based on Kyoto Encyclopedia of Genes and Genomes (KEGG).

### Real-time quantitative RT-PCR

Total RNA was isolated from the samples using Trizol reagent (Invitrogen) according to the manufacturer’s instructions. Any potential DNA contamination was removed by RNase-free DNase treatment (Promega). cDNA was prepared using the Transcriptor First Strand cDNA Synthesis Kit (Roche). Relative expression quantification was performed using the ABI PRISM 7500 Fast sequence detection system (Applied Biosystems, Foster City, CA, USA), which measures real-time SYBR Green fluorescence (Roche). Briefly, cDNA was mixed with 1 μL forward and reverse primers (5 μM of each), 8 μL RNase-free water, and 10 μL 2× PCR SYBR Green Mix buffer in a 20-μL reaction. Next, 40 cycles of PCR were conducted at 95 °C for 15 s, and 60 °C for 1 min within each cycle. Expression was quantified by the comparative Ct method (2^-ΔΔCt^) with *GAPDH* used as an internal control. Primers used in quantitative RT-PCR are listed in Supplementary File 1 (Supplemental Table S3).

### Immunofluorescence confocal microscopy

HeLa cells were transfected with shPRMT5, replated 24 h later onto coverslips and cultured overnight. The cells were washed with PBS, fixed in 4% paraformaldehyde, permeabilized with 0.2% Triton X-100, blocked with 5% normal goat serum (Sigma–Aldrich), and incubated with appropriate primary antibodies overnight followed by staining with TRITC-conjugated secondary antibodies for 1 h at room temperature. The cells were washed four times, and the nuclei were stained with 4, 6-diamidino-2-phenylindole (DAPI) at a final concentration of 0.1 μg/mL. Images were visualized and recorded with an Olympus FV1000 confocal microscope (Tokyo, Japan).

### Cell invasion assay

Transwell chamber filters (Chemicon Incorporation, Temecula, CA, USA) were coated with Matrigel. After infection with lentivirus, the cells were resuspended in serum-free media and 2.5 × 10^4^ cells in 0.5 mL serum-free media were placed in the upper chamber of the transwell. The chamber was then transferred to a well containing 500 μL of media containing 10% fetal bovine serum. The HeLa cells were incubated for 18 h and Ca Ski cells for 24 h at 37 °C. Cells in the upper well were removed by wiping the top of the membrane with cotton swabs. The membranes were then stained, and the remaining cells were counted. Three high-powered fields were counted for each membrane.

### Mouse xenograft models

HeLa cells that had been transfected to stably express firefly luciferase (Xenogen Corporation, Alameda, CA, USA) were infected with lentiviruses carrying shRNAs or overexpression constructs. These cells were subcutaneously implanted (5 × 10^6^ cells for shRNA group and 2.5 × 10^6^ cells for overexpression group) or injected into the lateral tail vein (2 × 10^6^ cells for shRNA group and 1 × 10^6^ cells for overexpression group) of 6-week-old female SCID mice following randomization. For bioluminescence imaging, the mice were injected abdominally with 200 mg/kg of D-luciferin in PBS. At 15 min after injection, the mice were anesthetized, and bioluminescence was imaged with a charge-coupled device camera (IVIS; Xenogen Corporation). Bioluminescence images were obtained with a 15-cm field of view, binning (resolution) factor of eight, 1/f stop, open filter, and imaging time of 30 s to 2 min. Bioluminescence from the relative optical intensity was defined manually. Photon flux was normalized to the background, which was defined from the relative optical intensity drawn over a mouse not injected with luciferin. No blinding was done.

### Tissue specimens and immunohistochemistry (IHC)

Samples were frozen in liquid nitrogen immediately after surgical removal and stored at −80 °C until analysis. The samples were fixed in 4% paraformaldehyde (Sigma–Aldrich) at 4 °C overnight and then embedded in paraffin, sectioned at 8 μm onto Superfrost-Plus Slides, processed by standard protocols using DAB staining, and monitored microscopically.

### Statistical analysis

Results are reported as the mean ± SD unless otherwise noted. Comparisons were performed using two-tailed unpaired *t*-test. SPSS V.17.0 software (SPSS, Inc., Chicago, IL, USA) was used for statistical analysis. Data are representative of at least three independent experiments. Tumor datasets were downloaded from http://www.ncbi.nlm.nih.gov/geo and GSE numbers are shown in the Figures. Data for Kaplan–Meier survival analysis were from http://kmplot.com/analysis.

## Results

### PRMT5 is upregulated in cervical cancer and is related to stemness maintenance of cervical cancer cells

**Fig. 1:**
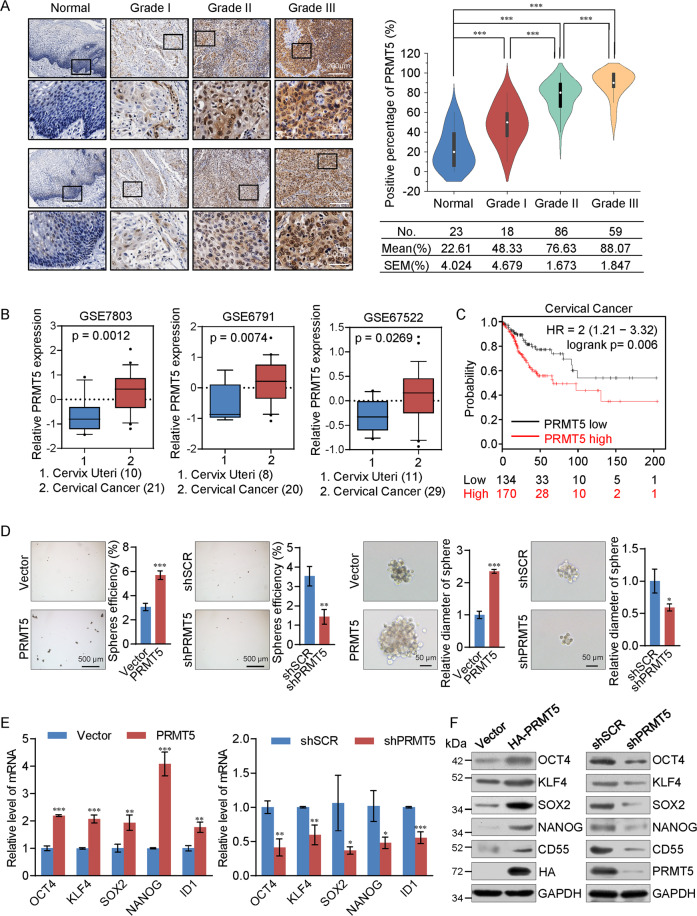
PRMT5 is upregulated in cervical cancer and promotes maintenance of stemness in cervical cancer cells. **A** Immunohistochemical staining of PRMT5 in normal cervical tissues and cervical cancers (histological grades I, II, and III). Positively stained nuclei (in percentages) in grouped samples were analyzed by two-tailed unpaired *t*-test (*p < 0.05, **p < 0.01, ***p < 0.001). **B** Analysis of public datasets (GSE7803, GSE6791, and GSE67522) for the expression of PRMT5 by two-tailed unpaired *t*-test (*p < 0.05, **p < 0.01, ***p < 0.001). **C** Kaplan–Meier survival analysis of the relationship between survival time and PRMT5 in cervical cancer using the online tool. **D** HeLa cells were transfected with the shRNA or expression construct of PRMT5 for spheroid-forming assays. The images represent one field under microscopy in each group. **E,** **F** Expression of the indicated stemness markers was measured by real-time RT-PCR (**E**) or western blotting (**F**) in HeLa cells, with spheroid cells in (**D**).

To investigate the role of PRMT5 in cervical cancer progression, we collected 186 cervical cancer samples and adjacent normal tissues and performed tissue microarrays with IHC staining to examine PRMT5 expression. PRMT5 was strikingly upregulated in cervical cancer and its expression was positively correlated with histological tumor grade (Fig. 1A).

Next, analysis of three published clinical datasets (GSE7803, GSE6791, and GSE67522) revealed that PRMT5 is highly expressed in cervical cancer cells than in adjacent normal tissues (Fig. 1B). Kaplan–Meier survival analysis showed that lower expression of PRMT5 was associated with improved survival of patients with cervical cancer (Fig. 1C).

To further investigate the role of PRMT5 in cervical cancer development, spheroid-forming assays were performed in HeLa cells. Spheroids were formed after 2 weeks in suspension culture medium. Overexpression of PRMT5 resulted in an increased sphere-forming rate and volume of cancer cell spheres, whereas knockdown of PRMT5 showed opposite result (Fig. 1D). Moreover, enhanced mRNA levels of “stemness” marker genes (*OCT4*, *KLF4*, *SOX2*, *NANOG*, and *ID1*) were detected in spheroid cells overexpressing PRMT5, while PRMT5 knockdown decreased them (Fig. 1E). The protein levels of these markers correlated with mRNA levels (Fig. 1F), indicating that PRMT5 is positively correlated with the stemness maintenance of cervical cancer cells.

### PRMT5 promotes EMT and invasion potential of cervical cancer cells in vitro and cervical cancer metastasis in vivo

**Fig. 2:**
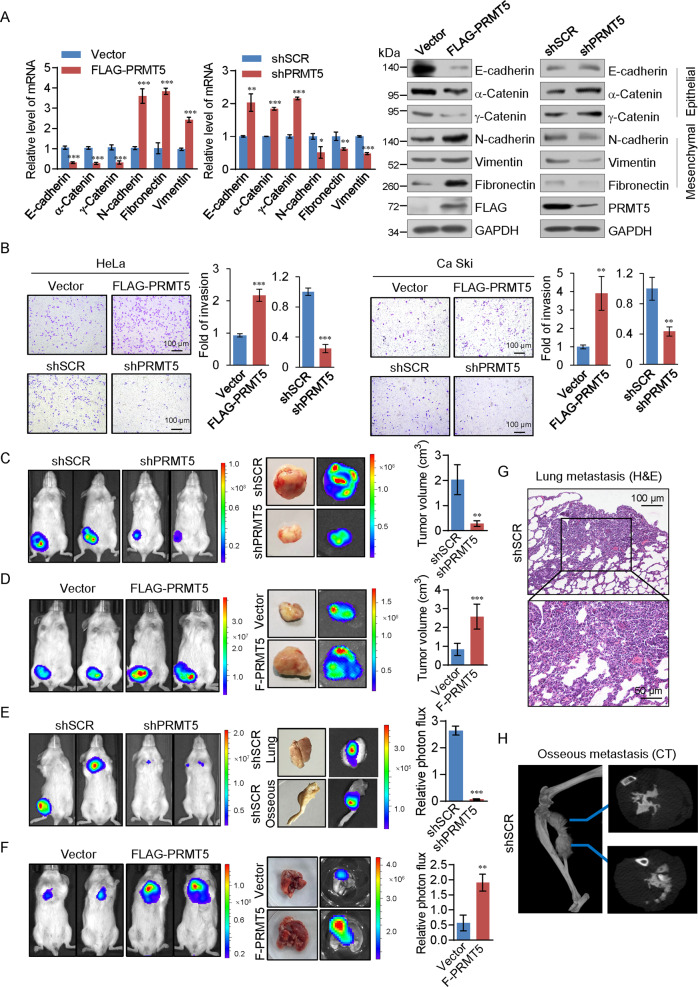
PRMT5 promotes EMT and the invasive potential of cervical cancer cells in vitro and cervical cancer metastasis in vivo. **A** Expression of the indicated epithelial or mesenchymal markers was measured by real-time RT-PCR or western blotting in HeLa cells with PRMT5 overexpression or depletion. **B** HeLa cells and Ca ski cells transfected with specific shRNA or expression constructs of PRMT5 were performed using Matrigel transwell filters. The invaded cells were stained and counted after 18 h for HeLa and 24 h for Ca ski cells. The images represent one field under microscopy in each group. **C, D** HeLa-Luc-D3H2LN cells infected with lentiviruses carrying specific shRNA or expression constructs of PRMT5 were inoculated subcutaneously into the abdomen of 6-week-old female SCID mice (*n* = 8). Primary tumors were quantified by bioluminescence imaging 6 weeks after initial implantation. Representative in vivo bioluminescent images are shown, and tumor specimens were examined by in vitro bioluminescent measurements. **E–H** HeLa-Luc-D3H2LN cells were injected intravenously through the tail vein of 6-week-old female SCID mice (*n* = 8). Lung metastases were quantified by bioluminescence imaging after 6 weeks. Representative in vivo bioluminescent images are shown. Metastases in lung tissue were stained with hematoxylin and eosin (H&E) (**G)** and metastases in osseous tissue were verified by microCT (**H**). **A–F** Two-tailed unpaired *t-*test was used (*p < 0.05, **p < 0.01, ***p < 0.001).

We next investigated the role of PRMT5 in the metastasis of cervical cancer. Gain-of-function and loss-of-function analyses of PRMT5 were performed with lentivirus delivering PRMT5 CDS or shRNAs, and the expression of epithelial/mesenchymal markers was analyzed in HeLa cells. We found that overexpression of PRMT5 resulted in the reduction, at both mRNA and protein levels, of epithelial protein markers including E-cadherin, α-Catenin, and γ-Catenin and the induction of mesenchymal markers including N-cadherin, Fibronectin, and Vimentin. Consistently, depletion of PRMT5 resulted in the opposite result (Fig. 2A).

We then investigated the roles of PRMT5 in the invasion potential using transwell assays. The results in HeLa and Ca Ski showed that overexpression of PRMT5 resulted in an increased invasive potential of cervical cancer cells, whereas knockdown of PRMT5 showed the opposite result (Fig. 2B). Furthermore, the viability of cells was measured by CCK8 assays which showed that the cell proliferation had no effect on the results at the specified time (Supplemental Fig. S1). These results support that PRMT5 plays a critical role in regulating EMT and promoting the invasive potential of cervical cancer.

To further investigate the role of PRMT5 in tumor development and progression in vivo, HeLa cells stably expressing firefly luciferase were then co-infected with specific shRNA or expression constructs of PRMT5. To measure orthotopic tumorigenesis or seeding metastasis, HeLa-Luc-D3H2LN cells were either implanted subcutaneously (*n* = 8) or injected into the lateral tail vein (*n* = 8) of 6-week-old female SCID mice. Tumor metastasis was measured after 6 weeks. In subcutaneously implanted groups, knockdown of PRMT5 resulted in a significant reduction in tumor growth (Fig. 2C), whereas overexpression of PRMT5 showed the opposite result (Fig. 2D). In the intravenous groups, PRMT5 knockdown led to a dramatic decrease in osseous metastasis and lung metastasis (Fig. 2E), and overexpression of PRMT5 resulted in an increase in lung metastasis (Fig. 2F). Lung tissue metastases were then verified by histological staining, while osseous tissue metastases were verified by microCT (Fig. 2G, H). The results indicated that PRMT5 promotes the tumorigenesis and metastasis of cervical cancer in vitro and in vivo.

### PRMT5 is physically associated with Snail and the NuRD(MTA1) complex

**Fig. 3:**
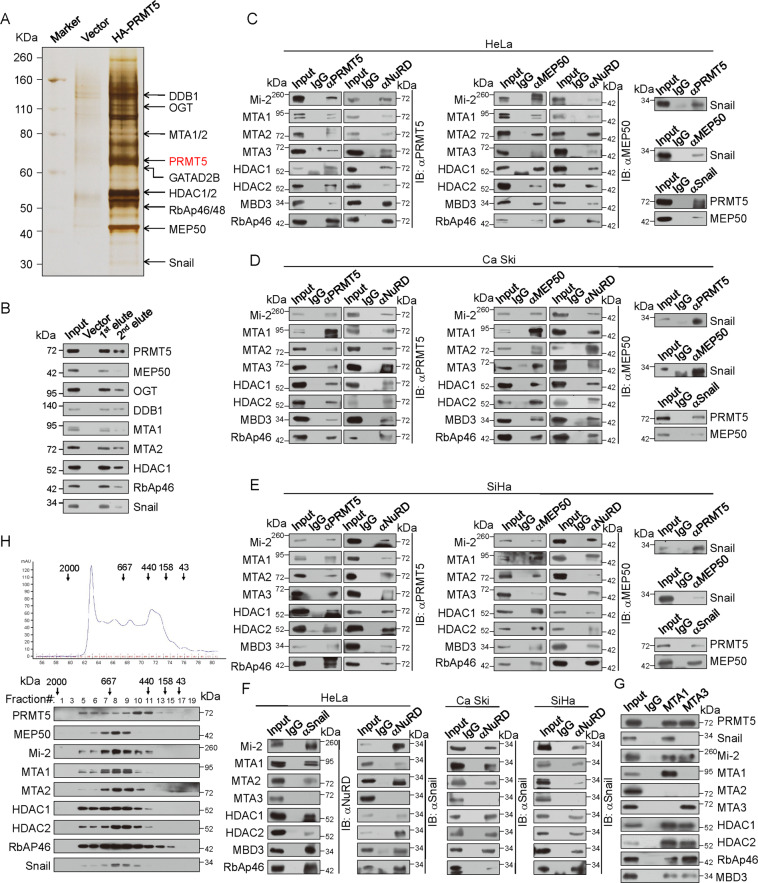
PRMT5 interacts with snail and the NuRD complex. **A** Immunoaffinity purification of PRMT5-containing protein complexes. Cellular extracts from HeLa cells stably expressing HA (control) or HA-PRMT5 were immunopurified with anti-HA affinity columns and eluted with HA peptide. The eluates were resolved by SDS-PAGE and silver stained. The protein bands were retrieved and analyzed by mass spectrometry. Detailed results of the mass spectrometric analysis are provided in Supplementary File 1 (Supplemental Table S4). **B** Western blotting analysis of purified fractions using antibodies against the indicated proteins. **C–E** Association of PRMT5 with Snail and the NuRD complex. Whole-cell lysates from HeLa, Ca ski, and SiHa cells were prepared and co-IP was performed with antibodies against the indicated proteins. Immunocomplexes were then tested by IB using antibodies against the indicated proteins. IgG served as a negative control. **F** Association of Snail with the NuRD complex. Whole-cell lysates from HeLa, Ca ski, and SiHa cells were prepared and co-IP was performed with antibodies against the indicated proteins. Immunocomplexes were then tested by IB using antibodies against the indicated proteins. **G** Equal amounts of HeLa cellular extracts were immunoprecipitated with antibodies against MTA1 or MTA3 followed by IB with antibodies against the indicated proteins. **H** Cofractionation of Snail, PRMT5, and the NuRD complex by FPLC. Nuclear extracts of HeLa cells were fractionated on a DEAE Sepharose column followed by a Superose 6 gel filtration column. The fractions were analyzed by western blotting. Molecular weight standards are shown on top (in kDa).

To better understand the mechanistic role of PRMT5, we conducted affinity purification and mass spectrometry to identify the proteins associated with PRMT5. HA-tagged PRMT5 (HA-PRMT5) was stably expressed in HeLa cells. Cellular extracts were subjected to affinity purification using an anti-HA affinity gel. Mass spectrometric analysis revealed that PRMT5 co-purified with MTA1, MTA2, GATAD2B, HDAC1, and RbAp46/48, which are components of the NuRD complex, as well as with MEP50, OGT, DDB1, and Snail (Fig. 3A). The detailed results are shown in Supplementary File 1 (Supplemental Table S4). Among the PRMT5 co-purified proteins, the association of PRMT5 with OGT and MEP50 has been previously reported [37]. The presence in the PRMT5 interactome was confirmed by western blotting with antibodies against the corresponding proteins (Fig. 3B).

To confirm the physical association of PRMT5 with the NuRD complex and Snail, co-immunoprecipitation (co-IP) experiments were performed with HeLa cells. Immunoprecipitation (IP) with antibodies against PRMT5 and MEP50 followed by immunoblotting (IB) with antibodies against the components of NuRD complex and Snail demonstrated that the PRMT5-MEP50 complex co-immunoprecipitated with them. Reciprocally, IP with NuRD subunits and Snail antibodies and IB using antibodies against PRMT5 or MEP50 confirmed these interactions (Fig. 3C). To further verify the association, Ca Ski and SiHa cervical cancer cells were used to perform the same co-IP experiments (Fig. 3D, E). These results suggest that the PRMT5-MEP50 complex is physically associated with the NuRD complex and Snail in vivo.

Based on the co-purification of Snail and the NuRD complex in the PRMT5 interactome and the functions of these components in cancer metastasis, co-IP experiments were performed using HeLa cells. The results demonstrated that all tested proteins, except for MTA3, efficiently co-immunoprecipitated with Snail. The results were also confirmed in Ca Ski and SiHa cells (Fig. 3F). These results suggest that Snail is physically associated with the MTA1− or MTA2− but not the MTA3-associated NuRD complex in vivo. Corroborating this result, IP using HeLa cellular extracts with antibodies against MTA1 or MTA3, followed by immunoblotting with antibodies against Snail, PRMT5, or NuRD components, indicated that, while PRMT5 co-immunoprecipitated with both MTA1 and MTA3, Snail did not co-immunoprecipitate with MTA3 (Fig. 3G).

To verify the formation of a Snail/PRMT5/NuRD complex, HeLa nuclear proteins were fractionated by FPLC using a high-salt extraction and size exclusion approach. We found that PRMT5 eluted with an apparent molecular mass much greater than that of the monomeric protein. Western blotting revealed a major peak at ~500–1500 kDa for PRMT5-MEP50, the NuRD complex proteins and Snail (Fig. 3H). Significantly, the chromatographic profiles of Snail, PRMT5, and the NuRD complex largely overlapped. These observations support the existence of a Snail/PRMT5/NuRD(MTA1) complex in vivo and suggest that these proteins function in a concerted manner.

### Molecular interaction between Snail, PRMT5, and the NuRD(MTA1) complex

**Fig. 4:**
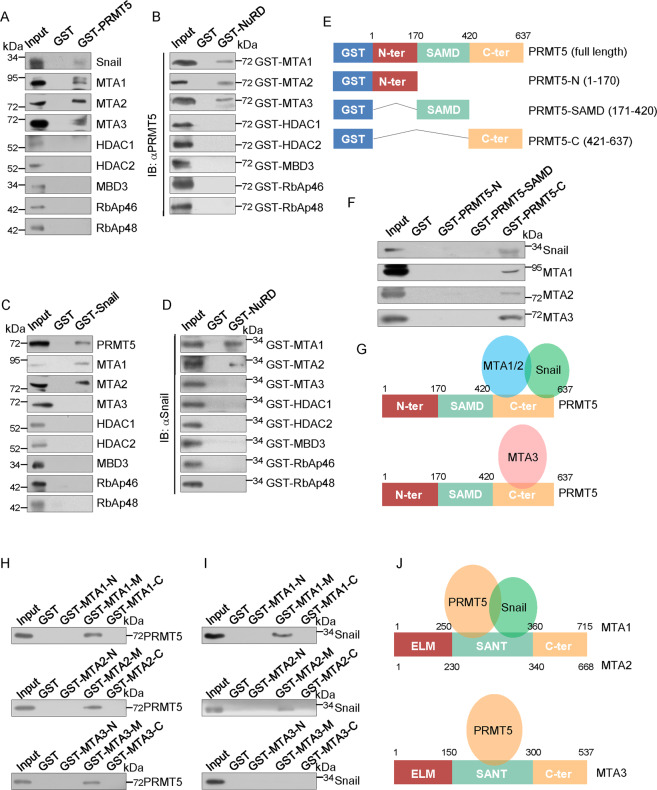
Molecular interaction between snail, PRMT5, and the NuRD complex subunits. **A–D** GST pull-down experiments with bacterially expressed GST-fused proteins and in vitro transcribed/translated indicated proteins. **E–G** Identification of essential domains required for the interaction with Snail or the NuRD complex of PRMT5. GST pull-down experiments with a bacterially expressed series of truncation vectors of PRMT5 (N, SAMD, and C) to generate GST fusion proteins and in vitro transcribed/translated indicated proteins. **H–J** Identification of essential domains required for the interaction with Snail or PRMT5 of MTAs. GST pull-down experiments with a bacterially expressed series of truncation vectors of MTAs (ELM, SANT, and C) to generate GST fusion proteins and in vitro transcribed/translated indicated proteins.

To further support the physical association between Snail, PRMT5, and the NuRD(MTA1) complex and gain insight into the molecular details, GST pull-down experiments were performed by incubation of GST-fused PRMT5 (GST-PRMT5) with in vitro transcribed/translated Snail and individual components of the NuRD complex. These experiments revealed that PRMT5 interacted directly with Snail, MTA1, MTA2, and MTA3 (Fig. 4A). Reciprocal GST pull-down experiments with GST-fused NuRD components and in vitro transcribed/translated PRMT5 yielded similar results (Fig. 4B). The following GST pull-down assays using GST-fused Snail, and in vitro transcribed/translated PRMT5 and subunits of the NuRD complex verified that Snail interacted directly with PRMT5, MTA1, and MTA2 but not with MTA3 (Fig. 4C). Reciprocal GST pull-down experiments with GST-fused NuRD components and in vitro transcribed/translated Snail verified these results (Fig. 4D).

Additionally, GST pull-down assays with the GST-fused N-terminal fragment (PRMT5-N), enzymatic domain (PRMT5-SAMD), or C-terminal fragment (PRMT5-C) of PRMT5 (Fig. 4E) and in vitro transcribed/translated Snail and MTAs indicated that the C-terminal region of PRMT5 is responsible for the interaction with Snail and MTAs (Fig. 4F, G). Moreover, GST pull-down assays with the GST-fused N-terminal fragment (ELM), middle region (SANT), or C-terminal fragment (C-ter) of MTAs and in vitro transcribed/translated PRMT5 or Snail suggested that the SANT domain is responsible for the interaction of MTA1/2/3 with PRMT5 and of MTA1/2 with Snail (Fig. 4H, I, and J).

Taken together, these results not only provide strong support for a physical association between Snail, PRMT5, and NuRD(MTA1), but also reveal the molecular details involved in the formation of the Snail/PRMT5/NuRD(MTA1) complex.

### Genome-wide identification of transcription targets for the Snail/PRMT5/NuRD(MTA1) complex

**Fig. 5:**
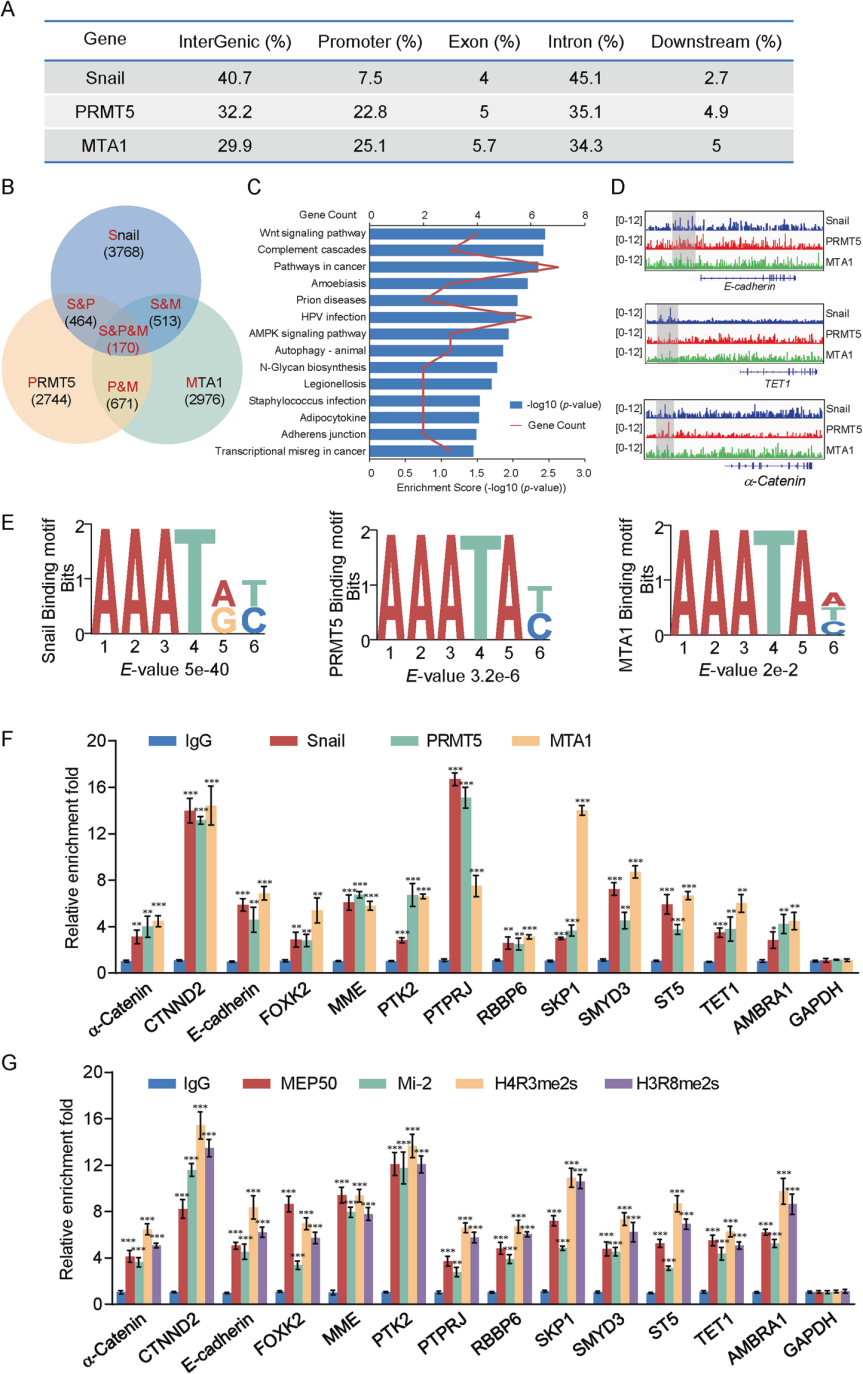
Genome-wide transcription target analysis of the snail/PRMT5/NuRD(MTA1) complex. **A** Genomic distribution of Snail, PRMT5, and MTA1 determined by ChIP-seq analysis. **B** Venn diagram of overlapping promoters bound by Snail, PRMT5, and MTA1 in HeLa cells. **C** Pathway analysis of the 170 overlapping target genes of PRMT5/Snail/MTA1 arranged into functional groups. **D** Visualized peaks at representative loci using an integrative genomics viewer. **E** Snail-, PRMT5-, and MTA1-bound motifs were analyzed using MEME suite. **F, G** Verification of ChIP-seq results by qChIP analysis of the indicated genes in HeLa cells. Results are represented as the fold-change compared to the control with glyceraldehyde 3-phosphate dehydrogenase (GAPDH) as a negative control. Error bars represent the mean ± SD for three independent experiments (*p < 0.05, **p < 0.01, ***p < 0.001, and two-tailed unpaired *t*-test).

To further investigate the functional significance of the Snail/PRMT5/NuRD(MTA1) complex, we analyzed the genome-wide transcriptional targets by chromatin IP-based deep sequencing (ChIP-seq). In these experiments, ChIP was conducted using HeLa cells with antibodies against Snail, PRMT5, and MTA1. Following ChIP, the DNA was amplified, labeled, and then sequenced (GEO accession number: GSE130194). Using a HiSeq2000 and a *p*-value cutoff of 10^−3^, we identified 4915 Snail-specific, 4049 PRMT5-specific, and 4330 MTA1-specific binding promoters (Fig. 5A). Data were then analyzed for overlapping DNA sequences/gene promoters. These experiments identified a total of 170 different promoters targeted by Snail, PRMT5, and MTA1 (Supplementary File 2). The corresponding genes to these promoters were classified into various cellular signaling pathways using KEGG pathway software (Fig. 5B, C). Importantly, we found that Snail, PRMT5, and MTA1 had similar peak locations on the proximal promoter of the target genes (Fig. 5D) and binding motifs (Fig. 5E), supporting that these proteins physically interact and are functionally linked.

Quantitative ChIP (qChIP) analysis in HeLa cells using specific antibodies against Snail, PRMT5, MTA1, MEP50, Mi-2, H4R3me2s, H3R8me2s, or IgG showed that the promoters of these genes were strongly enriched, including *E-cadherin*, *α-Catenin*, *WDR94*, *SMYD3*, *FOXK2*, *MME*, *PTK2*, *PTPRJ*, *SKP1*, *CTNND2*, *ST5*, *TET1*, and *RBBP6*, validating the ChIP-seq results (Figs. 5F, G).

### Formation of the Snail/PRMT5/NuRD(MTA1) repression complex on transcriptional targets

**Fig. 6:**
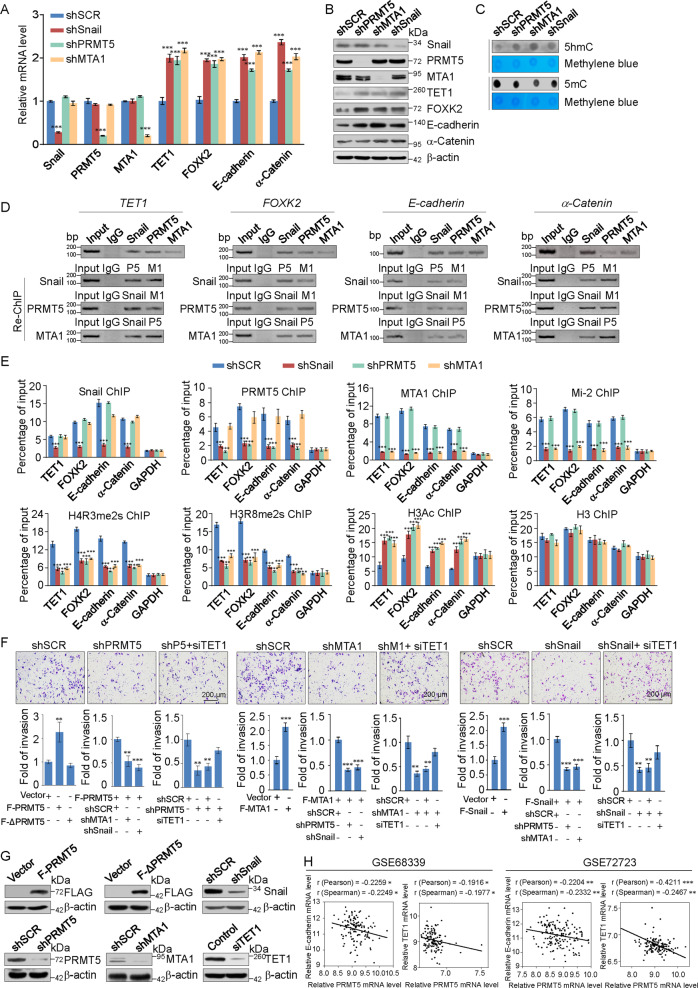
Formation of snail/PRMT5/NuRD(MTA1) repression complex on transcriptional targets. **A, B** Clones in which Snail, PRMT5, and MTA1 stably knocked down were compared to the parental cell line with respect to mRNA and protein levels of indicated genes in HeLa cells. The mRNA levels were normalized to those of GAPDH and β-actin served as a loading control for western blotting. Error bars represent the mean ± SD of three independent experiments. (*p < 0.05, **p < 0.01, ***p < 0.001, and two-tailed unpaired *t*-test). **C** Equal amounts of genomic DNA from HeLa cells were used for dot blot assays with antibodies against 5mC or 5hmC. **D** ChIP and Re-ChIP experiments were performed in HeLa cells with the indicated antibodies. **E** HeLa cells were infected with lentiviruses carrying the indicated shRNAs. qChIP analysis of the selected promoters was performed using antibodies against Snail, PRMT5, MTA1, Mi-2, H4R3me2s, H3R8me2s, or H3Ac. H3 was detected as an internal control. Results are represented as the fold-change over the control with GAPDH as a negative control. **F, G** HeLa cells were transfected with the indicated specific shRNAs or/and expression constructs for cell invasion assays. The invaded cells were stained and counted. The images represent one field under microscopy in each group. The efficiency of protein knockdown or overexpression was verified by western blotting. F-∆PRMT5, PRMT5 expression construct without the SAMD domain (enzymatic domain). **H** Analysis of public datasets (GSE68339 and GSE72723) for the expression of PRMT5 and E-cadherin or TET1 in cervical cancer. The relative levels of E-cadherin and TET1were plotted against that of PRMT5.

To further support that Snail, PRMT5, and MTA1 occupy the target promoters, HeLa cells were infected with lentivirus-delivered shRNA targeted to *Snail*, *PRMT5*, and *MTA1* mRNA along with a scrambled shRNA control. The shRNAs significantly reduced the expression of their target genes without causing detectable changes in non-targeted genes. Additionally, knockdown of Snail, PRMT5, or MTA1 led to increased expression of *TET1*, *FOXK2*, *E-cadherin*, and *α-Catenin* at both the transcriptional and protein levels (Fig. 6A, 6B). As TET1 is a methylcytosine dioxygenase that converts 5-methylcytosine (5mC) to 5-hydroxymethylcytosine (5hmC), dot blot assays found that depletion of Snail, PRMT5 or MTA1 resulted in a reduction in 5mC and increase in 5hmC (Fig. 6C).

We next investigated the regulation of target genes by the Snail/PRMT5/NuRD(MTA1) complex. We demonstrated that Snail, PRMT5, and MTA1 co-occupied the promoters of *TET1*, *FOXK2*, *E-cadherin*, and *α-Catenin* through ChIP assays. To further support that Snail, PRMT5, and MTA1 function in the same protein complex at the promoters of target genes, ChIP/Re-ChIP experiments were performed on the representative target genes [28]. Soluble chromatin was first immunoprecipitated with antibodies against Snail, PRMT5, or MTA1. The immunoprecipitates were subsequently reimmunoprecipitated with appropriate antibodies. The results showed that the *TET1*, *FOXK2*, *E-cadherin*, and *α-Catenin* promoters were co-immunoprecipitated with antibodies against Snail, PRMT5, or MTA1 (Fig. 6D). These results support that Snail, PRMT5, and the NuRD(MTA1) complex occupy the target promoters in one protein complex.

To identify the functional association between Snail, PRMT5, and MTA1 on target promoters, HeLa cells with Snail, PRMT5, or MTA1 stably depleted were used. qChIP experiments with antibodies against these proteins indicated that depletion of one of the proteins resulted in marked reduction in the recruitment of corresponding proteins at the target promoters. Depletion of Snail also dramatically reduced the recruitment of PRMT5, MTA1, and Mi-2. Subsequent qChIP experiments showed that the levels of H4R3me2s and H3R8me2s were significantly decreased at target promoters upon depletion of Snail, PRMT5, or MTA1, while the level of H3Ac was markedly increased and the level of H3 did not change (Fig. 6E). These results indicate that PRMT5 and NuRD(MTA1) are recruited by Snail to the target gene promoters, supporting that the Snail/PRMT5/NuRD(MTA1) complex forms on target gene promoters and represses transcription.

As described above, our genome-wide analysis indicated that the Snail/PRMT5/NuRD(MTA1) complex play important roles in cell migration and tumor cell invasion. Based on the roles of PRMT5, Snail, and MTA1 in EMT and cancer progression [38, 39], we then further identified the functional coordination of the Snail/PRMT5/NuRD(MTA1) complex on target promoters using transwell assays. Overexpression of Snail, PRMT5, or MTA1 led to increased invasive potential of HeLa cells, whereas knockdown showed the opposite result. Importantly, confirming that Snail/PRMT5/NuRD(MTA1) functions as a complex, the positive effect of overexpression of Snail, PRMT5, or MTA1 on the invasive potential was offset by transfecting with shRNAs target to other proteins. Additionally, the inhibitory effect of Snail, PRMT5, or MTA1 knockdown was partially offset by TET1 knockdown (Fig. 6F). The efficiency of protein knockdown or overexpression was verified by western blotting (Fig. 6G). Moreover, analysis of published clinical datasets (GSE68339 and GSE72723) revealed a significantly negative correlation between the expression of TET1, E-cadherin, and expression of PRMT5, supporting that TET1 and E-cadherin are transcriptionally repressed by PRMT5 (Fig. 6H). These results support that the Snail/PRMT5/NuRD(MTA1) complex plays a critical role in regulating EMT and promoting the migration and invasive potential of cervical cancer cells.

### PRMT5 inhibitor EPZ015666 suppresses tumor progression of cervical cancer cells

**Fig. 7:**
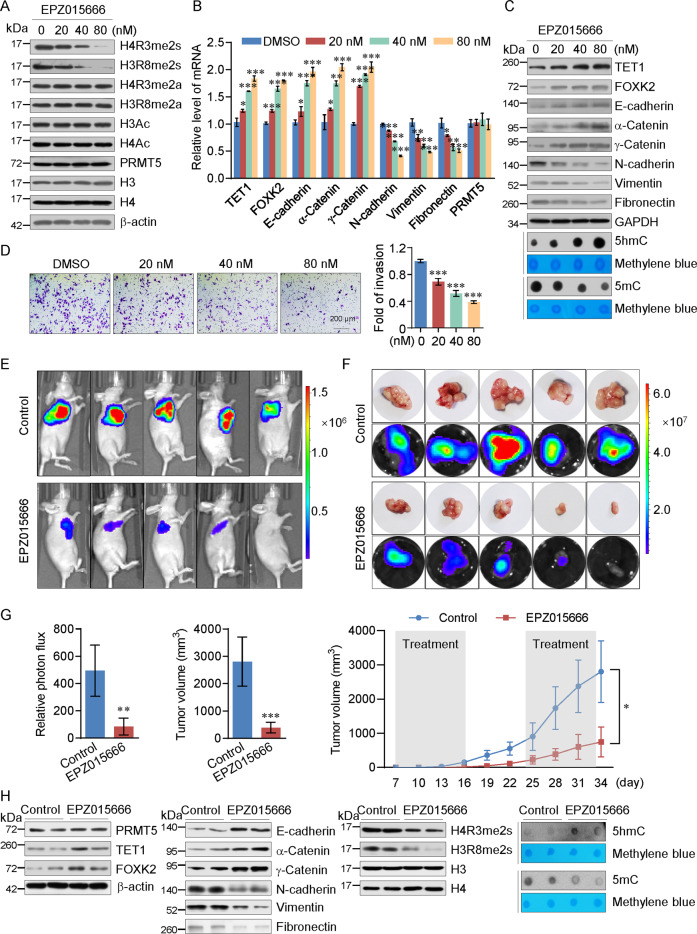
PRMT5 inhibitor EPZ015666 suppresses tumor progression of cervical cancer cells. **A** Western blotting analysis using HeLa cells treated with different concentrations of EPZ015666 with antibodies against the indicated proteins. **B, C** Expression of the indicated proteins was measured by real-time RT-PCR or western blotting in HeLa cells treated with different concentrations of EPZ015666. **D** HeLa cells were treated with different concentrations of EPZ015666 for cell invasion assays. The invaded cells were stained and counted. The images represent one field under microscopy in each group. Each bar represents the mean ± SD for triplicate experiments (*p < 0.05, **p < 0.01, ***p < 0.001). **E–G** HeLa-Luc-D3H2LN cells were inoculated subcutaneously into 6-week-old female nude mice, which were then treated with EPZ015666 or vehicle. Tumors were quantified by bioluminescence imaging after two 10-day periods. Tumor growth of xenograft nude mice treated with EPZ015666 or vehicle was shown in the right (*n* = 5, mean ± SD). Tumor specimens were examined by in vitro bioluminescent measurements. Two-tailed unpaired *t*-test (*p < 0.05, **p < 0.01, ***p < 0.001). **H** Western blot results showed expression of the indicated proteins measured in tumor tissue samples obtained from xenograft nude mice. DNA dot blot analysis showed genomic 5mC and 5hmC level of tumor tissue and methylene blue staining served as loading control (right).

EPZ015666 (GSK3235025) is an orally bioavailable small-molecule inhibitor of PRMT5 enzymatic activity in the nanomolar range [40]. We next investigated the role of EPZ015666 in the metastasis of cervical cancer. We found that EPZ015666 inhibited the enzymatic activity of PRMT5 without affecting the level of PRMT5 in a concentration-dependent manner in HeLa cells (Fig. 7A). The effects of EPZ015666 on EMT of cervical cancer cells were evaluated by RT-PCR and western blotting of whole-cell lysates from HeLa cells after 96 h. We found that EPZ015666 led to an induction in the expression of the target genes of PRMT5 and epithelial markers and reduction of mesenchymal markers at both the mRNA and protein levels in a concentration-dependent manner (Fig. 7B, C). Transwell assays showed that treatment with EPZ015666 decreased the invasive potential of cervical cancer cells (Fig. 7D). We next assessed the in vivo impact of EPZ015666. We first subcutaneously injected HeLa-Luc-D3H2LN cells into 6-week-old female nude mice (*n* = 5). After 7 days, we treated the mice with EPZ01566 (200 mg/kg, bid) or vehicle (0.5% MC) for two 10-day periods. We found that treatment of EPZ015666 resulted in a significant reduction in tumor growth compared to control (Fig. 7E-G). Then we used tumor tissue samples from xenograft nude mice to analyze the indicated proteins or genomic 5mC and 5hmC level by western blot or dot blot, and got similar results to in vitro experiments (Fig. 7H).

### Expression of PRMT5 is upregulated in multiple carcinomas and is a potential cancer biomarker

**Fig. 8:**
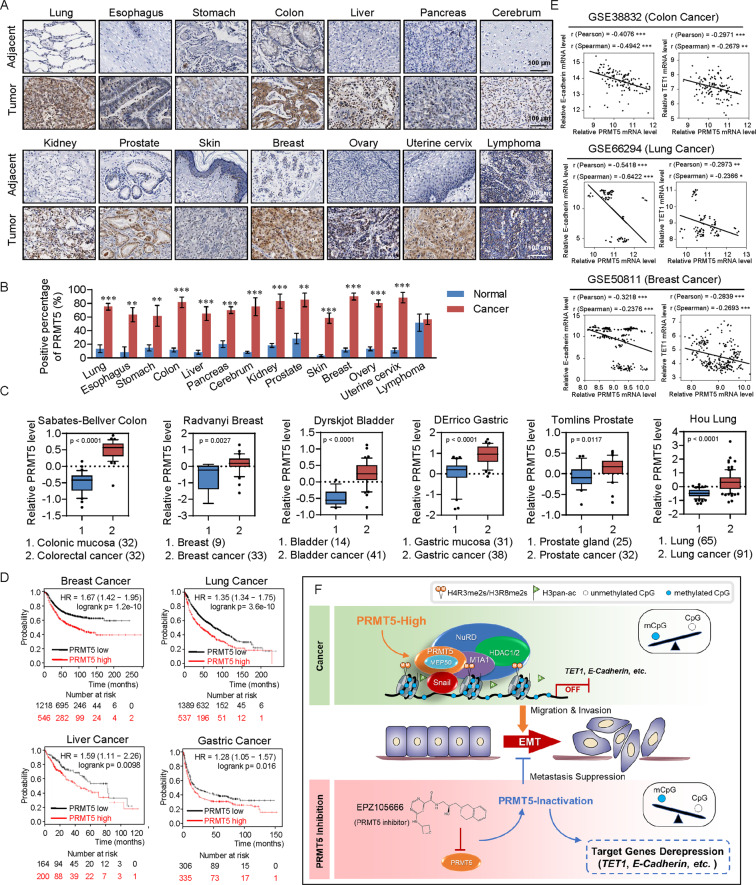
The expression of PRMT5 is upregulated in multiple carcinomas and is a potential cancer biomarker. **A, B** PRMT5 is upregulated in multiple carcinomas. Immunohistochemical staining of PRMT5 in paired samples of lung, esophagus, stomach, colon, liver, pancreas, cerebrum, kidney, prostate, skin, breast, ovary, uterine cervix, and lymphoma versus adjacent normal tissue. Representative images of 200-fold magnification of each type of paired tumor section are presented. **C** PRMT5 expression in multiple cancer microarray datasets available from Oncomine (https://www.oncomine.com/). **D** Kaplan–Meier survival analysis of the relationship between survival time and PRMT5 signature in breast, lung, liver, and gastric cancer using the online tool. **E** Analysis of published clinical datasets (GSE50811, GSE66294, and GSE38832) for the expression of TET1, E-cadherin, and expression of PRMT5 by two-tailed unpaired *t*-test (*p < 0.05, **p < 0.01, ***p < 0.001). **F** Graphic model as discussed in the text. DNA (black line); unmethylated CpG sites (hollow circle); methylated CpG sites (blue circle); H4R3me2s and H3R8me2s (orange ball); pan-acetylated H3 (green flag).

To investigate whether the effect of PRMT5 is observed in other types of cancers, we collected a series of carcinoma samples from patients. Tissue microarray analysis by IHC staining revealed significant upregulation of PRMT5 expression in carcinomas from multiple tissues (Fig. 8A, B). Furthermore, analysis of datasets from the Oncomine database (https://www.oncomine.com/) revealed high PRMT5 expression in many cancer types compared to adjacent normal tissues (Fig. 8C). Kaplan–Meier survival analysis revealed that lower expression of PRMT5 was associated with improved survival of patients with breast cancer, lung cancer, liver cancer, and gastric cancer (Fig. 8D). Moreover, analysis of published clinical datasets (GSE50811, GSE66294, and GSE38832) revealed a significantly negative correlation between the expression of TET1, E-cadherin, and expression of PRMT5, supporting that TET1 and E-cadherin are transcriptionally repressed by PRMT5 in multiple carcinomas (Fig. 8E). In summary, our analysis showed that PRMT5 is upregulated in multiple carcinomas and is a potential cancer biomarker.

## Discussion

Our results revealed that PRMT5 enables Snail and the NuRD(MTA1) complex to perform transcriptional silencing of a cohort of target genes, such as *TET1*, *FOXK2*, *E-cadherin*, and *α-Catenin*. Additionally, PRMT5 promotes the invasion and metastasis of cervical cancer in vitro and in vivo and its expression is markedly upregulated in multiple human cancers. The PRMT5 inhibitor EPZ015666 suppressed EMT and the invasive potential of cervical cancer cells, and led to an induction of *TET1* expression and 5hmC. Our data indicate that PRMT5 promotes tumorigenesis, progression, and metastasis, suggesting that it is a potential therapeutic target for cancer treatment (Fig. 8F).

Snail has been reported to interact with several corepressor complexes [23, 25, 41, 42]. In addition, the interaction between PRMT5 and Snail has been previously reported [43]. The study identified PRMT5 as a repressor recruited to the Snail complex via interaction with the AJUBA corepressor, but it is not clear whether there is a direct interaction between PRMT5 and Snail. We found that Snail interact with NuRD(MTA1) but not NuRD(MTA3). Similar to our previous findings showing that GATA3 and SIX3 selectively bind to NuRD(MTA3) [29, 44], we demonstrated the specificity between Snail and MTA1. Thus, the preference of Snail for transcription regulation factors appears to be ubiquitous and vital, suggesting that the preference for transcription factors is important in EMT and tumor development, providing insights useful for clinical diagnosis and drug design.

We found that Snail recruited PRMT5 and NuRD(MTA1) to form a transcriptional repression unit. The NuRD/HDAC1/2 complex is thought to be primarily involved in transcription repression based on its histone deacetylase activity [45]. We found that, catalytic activities of PRMT5 and NuRD(MTA1) complex were interdependent. PRMT5 and the NuRD complex may act in a coordinated manner to simultaneously methylate H4R3 or H3R8 and deacetylate histone, which are linked to transcription repression. The complex inhibited the expression of various transcription factors including *FOXK2*, which are known to be critically involved in EMT [13, 46]. Interestingly, the Snail/PRMT5/NuRD(MTA1) complex also inhibited the expression of E-cadherin and α-Catenin, which are molecular markers of EMT [46]. Thus, the Snail/PRMT5/NuRD(MTA1) complex may affect cervical epithelial plasticity by regulating the hierarchical molecular network of EMT.

Hypermethylation of the promoter region is the most common cause of tumor suppressor genes inactivation in tumors. There is a dynamic balance between promoter methylation catalyzed by the DNA methyltransferases (DNMTs) and demethylation catalyzed by the Tet methylcytosine dioxygenases [47]. Interestingly, TET1 was also a repression target of the Snail/PRMT5/NuRD(MTA1) complex. It has been reported that TET1 initiates demethylation of DNA and is a tumor suppressor gene with loss of function mutation or low expression in many malignant tumors [48, 49]. Loss of TET1 induces EMT and metastasis in many kinds of cancer [50–52]. TET1 can protect the promoter of E-cadherin from being methylated, thus promoting its expression and affecting the EMT process [53, 54]. We found that deletion of Snail, PRMT5, or MTA1 reduced 5mC, which is possibly through TET1 regulation. The inhibition of TET1 leads to abnormal hypermethylation of the tumor suppressor genes, which results in an increase of tumor malignancy and stemness.

The importance of arginine methyltransferase PRMT5 in tumorigenesis and embryonic development has been reported in several studies. Increased expression of PRMT5 have been observed in a wide range of human malignancies [55] [56–58]. A previous study showed that *Prmt5*^−/−^ murine models suffer early embryonic lethality and are incapable of producing embryonic stem (ES) cells [59]. We found that expression of PRMT5 was upregulated in cervical cancer and multiple carcinomas. Further analysis indicated that overexpression of PRMT5 promotes EMT and the invasive potential of cervical cancer cells. Thus, PRMT5 is a potential cancer biomarker.

Over the last decades, cancer stem cells (CSCs) have emerged as a “bad seed” in the pathogenesis of cancer and are a subpopulation of cancer cells that can initiate, maintain, and regenerate within the tumor bulk [60, 61]. Increasing evidence has indicated that CSCs not only drive tumorigenesis, but also are responsible for tumor metastasis, recurrence, and resistance to radiotherapy and chemotherapy in various tumors [62]. Recent data suggest that CD55 is upregulated in cervical sphere cells [63]. PRMT5 was positively correlated with the stemness maintenance of cervical cancer cells, suggesting that PRMT5 is a potential target of CSCs to overcome therapy resistance.

EPZ015666 is an orally bioavailable small-molecule inhibitor of PRMT5. Interestingly, a study of mantle cell lymphoma (MCL) showed that EPZ015666 leads to tumor cell death [40]. The latest studies suggest that EPZ015666 inhibits growth of malignant glioma and multiple myeloma cells [64, 65]. We confirmed that EPZ015666 inhibits the enzymatic activity of PRMT5 without affecting its expression level. Further, our results showed that EPZ015666 significantly inhibits EMT and metastasis of cervical cancer cells. More importantly, EPZ015666 promotes the conversion of 5mC to 5hmC in cervical cancer cells, which may depend on the inhibitory effect of PRMT5 on TET1 expression. These results suggest that EPZ015666 is a potential treatment for tumor cell growth and metastasis of cervical cancer, prompting further studies in in vivo models, such as patient-derived xenograft (PDX) models.

In summary, we identified the new transcriptional silencing complex Snail/PRMT5/NuRD(MTA1). We demonstrated that PRMT5 promotes the invasion and tumorigenesis of cervical cancer in vitro and in vivo. Our data indicate that PRMT5 promotes tumorigenesis, progression, and metastasis and suggest that PRMT5 is a potential therapeutic target for cancer treatment.

## Supplementary information

Supplementary File 1

Supplementary File 2
