# Supplementary material for: Snail/PRMT5/NuRD complex contributes to DNA hypermethylation in cervical cancer by TET1 inhibition
Source: Cell Death Differ. 2021 May 5;28(9):2818–36. doi: 10.1038/s41418-021-00786-z (PMC8408166; doi:10.1038/s41418-021-00786-z)
Supplement: Supplementary file 1 [file 41418_2021_786_MOESM1_ESM.docx]

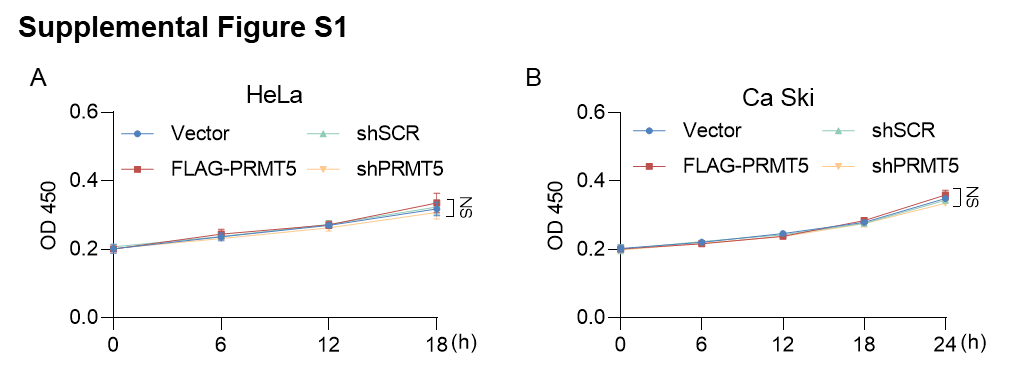


**Supplemental Figure S1. Cell proliferation had no effect on the transwell results at the specified time.**

The viability of HeLa (A) or Ca Ski (B) cells transfected with specific shRNA or expression constructs of PRMT5 was measured by CCK8 assay. NS, not significant.

**Supplemental Table S1.** The shRNA sequences used in this study were listed as following and * indicates the shRNA chosen for further study:

| shPRMT5＃1 | 5′- GGGACTGGAATACGCTAATTG-3′ * |
| --- | --- |
| shPRMT5＃2 | 5′-GGAATCTCAGACATATGAAGT-3′ |
| shPRMT5＃3 | 5′-GCTATTGCACCTTGGAATTTC-3′ |
| shMTA1＃1 | 5′-GGCTAACTTATTCCGAGAATG-3′ * |
| shMTA1＃2 | 5′-CCATACCTGATCCGGAGAATC-3′ |
| shMTA1＃3 | 5′-TGAAGCTGAGAGCAAGTTAAA-3′ |
| shSnail＃1 | 5′-GCAAATACTGCAACAAGGAAT-3′ * |
| shSnail＃2 | 5′-GGTACAACAGACTATGCAA-3′ |
| siTET1 | 5′-CAGUUAACUGAUCACGUGA-3′ |
| shSCR | 5′-TTCTCCGAACGTGTCACGT-3′ * |

**Supplemental Table S2.** The primers used in ChIP assays were listed as following:

| Gene | Strand | Sequence |
| --- | --- | --- |
| α-Catenin | F | 5′-TAGAGGGTAAACACGGTAAGGG-3′ |
| α-Catenin | R | 5′-GCTCACGGGATGATGAATAAG-3′ |
| E-cadherin | F | 5′-GAACCCTCAGCCAATCAGC-3′ |
| E-cadherin | R | 5′-CTGACTTCCGCAAGCTCACA-3′ |
| *SYMD3* | F | 5′-GACGAGCAGAAATGGCAAAGT-3′ |
| *SYMD3* | R | 5′-CGCATACGGAACAGTTGAAAA-3′ |
| *FOXK2* | F | 5′-CTCCTGACCTTGTGATCCACC-3′ |
| *FOXK2* | R | 5′-AAAAGAATTTCGCCACCGTTT-3′ |
| *MME* | F | 5′-TATTTTTCTCTTTCTCACTCCCC-3′ |
| *MME* | R | 5′-CTCTTTCTTCCCTTTCTTTTCCT-3′ |
| *PTK2* | F | 5′-TGGTCGGCTGTATCAATGAGGT-3′ |
| *PTK2* | R | 5′-AGGTTGGTGTTTTTCTGGGTTT-3′ |
| *PTPRJ* | F | 5′-AATGCGTCTCCAGAGTTCACAG-3′ |
| *PTPRJ* | R | 5′-AGCCCAGTACAAGCCAGTCC-3′ |
| *SKP1* | F | 5′-CGCTGTAGTGGCTTCGTCTTC-3′ |
| *SKP1* | R | 5′-ACTCACGGTGTTCGGTGTTAA-3′ |
| *CTNND2* | F | 5′-TGGGGTAGGAGGAATAAAAGC-3′ |
| *CTNND2* | R | 5′-AAGAGTGAAGGAGAAAAGCGG-3′ |
| *ST5* | F | 5′-ATCCAAATGTGGTCCATCAGC-3′ |
| *ST5* | R | 5′-CCAACCCTTAGTGCTTCCTTC-3′ |
| *RBBP6* | F | 5′-TTTTCTGGCAGGATGGTGATT-3′ |
| *RBBP6* | R | 5′-TAGCAGAGTTTCCCAGGGACTT-3′ |
| *TET1* | F | 5′-AAGTTTGACCTGGAAGGAATGAA-3′ |
| *TET1* | R | 5′-TTAAGACTGACTAGGGCATCTGAAT-3′ |
| *GAPDH* | F | 5′-CCCATACGACTGCAAAGACCC-3′ |
| *GAPDH* | R | 5′-AGCCACATCGCTCAGACACC-3′ |

**Supplemental Table S3.** The primers used in RT-PCR were listed as following:

| Gene | Strand | Sequence |
| --- | --- | --- |
| *PRMT5* | F | 5′-CTGTCTTCCATCCGCGTTTCA-3′ |
| *PRMT5* | R | 5′-GCAGTAGGTCTGATCGTGTCTG-3′ |
| E-cadherin | F | 5′-ATTTTTCCCTCGACACCCGAT-3′ |
| E-cadherin | R | 5′-TCCCAGGCGTAGACCAAGA-3′ |
| α-Catenin | F | 5′-ATGATCCCTGCTCTTCTGTG-3′ |
| α-Catenin | R | 5′-GATACCATCTTCCACAACTTTCAG-3′ |
| γ-Catenin | F | 5′-TGATGTGGGAAGACAGGCG-3′ |
| γ-Catenin | R | 5′-AGGAAGTCAGGAAGCAGGAAG-3′ |
| Fibronectin | F | 5′-GATAAATCAACAGTGGGAGCGG-3′ |
| Fibronectin | R | 5′-GTCTCTTCAGCTTCAGGTTTACTC-3′ |
| N-cadherin | F | 5′-AGTGCTGTTTTACCCCCTTAAA-3′ |
| N-cadherin | R | 5′-CTGATCCCTTTCAAATTCTTGC-3′ |
| Vimentin | F | 5′-GTAAGTACTCAGCTCTGCTCGT-3′ |
| Vimentin | R | 5′-CGGTCACCTTAACCTTTCTA-3′ |
| *Snail* | F | 5′-TCTAATCCAGAGTTTACCTTCCAG-3′ |
| *Snail* | R | 5′-CTGAAGTAGAGGAGAAGGACGA-3′ |
| *MTA1* | F | 5′-CCGAGAAACTAAAGCACCAG-3′ |
| *MTA1* | R | 5′-AAGAAGAAATCCTCCCGCTC-3′ |
| *TET1* | F | 5′-CCAACCTTAGGGAGTAACACTG-3′ |
| *TET1* | R | 5′-GGGAGTGCTGCTTCTTTCTG-3′ |
| *GAPDH* | F | 5′-GGAGCGAGATCCCTCCAAAAT-3′ |
| *GAPDH* | R | 5′-GGCTGTTGTCATACTTCTCATGG-3′ |

**Supplemental Table S4. Mass Spectrometry Analysis of PRMT5-containing Protein Complex**

| **Identified proteins** | **Band** | **Peptide number** | **Coverage** | **Peptides** |
| --- | --- | --- | --- | --- |
| DDB1 | 127kDa | 14 | 11.6% | IGRPSETGIIGIIDPEcR |
|  |  |  |  | ALYYLQIHPQELR |
|  |  |  |  | LPSFELLHK |
|  |  |  |  | QSGESIDIITR |
|  |  |  |  | QGQGQLVTcSGAFK |
|  |  |  |  | VTLGTQPTVLR |
|  |  |  |  |  |
| OGT | 103kDa | 3 | 2.3% | LVSIVADQLEK |
|  |  |  |  | EQGNIEEAVR |
|  |  |  |  | IIFSPVAPK |
|  |  |  |  |  |
| MTA2 | 75kDa | 6 | 8.8% | QIDQFLVVAR |
|  |  |  |  | EFEEESKQPGVSEQQR |
|  |  |  |  | TLLADQGEIR |
|  |  |  |  | TPTQLEGATR |
|  |  |  |  | QPSLHMSAAAASR |
|  |  |  |  |  |
| PRMT5 | 72kDa | 37 | 51.5% | LHNFHQLSAPQPcFTFSHPNRDPMIDNNR |
|  |  |  |  | LHNFHQLSAPQPcFTFSHPNR |
|  |  |  |  | cLLDRVPEEEKDTNVQVLmVLGAGR |
|  |  |  |  | VPLVAPEDLRDDIIENAPTTHTEEYSGEEK |
|  |  |  |  | VPEEEKDTNVQVLMVLGAGR |
|  |  |  |  | KVWYEWAVTAPVcSAIHNPTGR |
|  |  |  |  | DDIIENAPTTHTEEYSGEEK |
|  |  |  |  | VPEEEKDTNVQVLmVLGAGR |
|  |  |  |  | EKDRDPEAQFEMPYVVR |
|  |  |  |  | lHNFHQLSAPQPcFTFSHPNR |
|  |  |  |  | DRDPEAQFEMPYVVR |
|  |  |  |  | eKDRDPEAQFEMPYVVR |
|  |  |  |  |  |
| GATAD2B | 65kDa | 7 | 18.0% | TPVVQNAASIVQPSPAHVGQQGLSK |
|  |  |  |  | LQQQAALSPTTAPAVSSVSK |
|  |  |  |  | LPSRPGAQGVEPQNLR |
|  |  |  |  | ALQQEQEIEQR |
|  |  |  |  |  |
| HDAC1/2 | 55kDa | 4 | 8.1% | SIRPDNMSEYSK |
|  |  |  |  | YGEYFPGTGDLR |
|  |  |  |  | LFENLR |
|  |  |  |  | YHSDEYIK |
|  |  |  |  |  |
| RbAp48 | 48kDa | 5 | 10.3% | TPSSDVLVFDYTK |
|  |  |  |  | EMFEDTVEER |
|  |  |  |  | YMPQNPHIIATK |
|  |  |  |  | TVALWDLR |
|  |  |  |  |  |
| RbAp46 | 46kDa | 5 | 19.5% | IGEEQSPEDAEDGPPELLFIHGGHTAK |
|  |  |  |  | HPSKPDPSGEcNPDLR |
|  |  |  |  | TPSSDVLVFDYTK |
|  |  |  |  | YMPQNPcIIATK |
|  |  |  |  | TVALWDLR |
|  |  |  |  |  |
| MEP50 | 42kDa | 14 | 24.6% | AHAAQVTcVAASPHKDSVFLScSEDNR |
|  |  |  |  | YEHDDIVSTVSVLSSGTQAVSGSK |
|  |  |  |  | AHAAQVTcVAASPHK |
|  |  |  |  | YRSDGALLLGASSLSGR |
|  |  |  |  | aHAAQVTcVAASPHK |
|  |  |  |  | SDGALLLGASSLSGR |
|  |  |  |  | KETPPPLVPPAAR |
|  |  |  |  | sDGALLLGASSLSGR |
|  |  |  |  | FcKYEHDDIVSTVSVLSSGTQAVSGSK |
|  |  |  |  |  |
| Snail | 29kDa | 3 | 11.3% | PPPEILNPTASL |
|  |  |  |  | EAEAYAAFPG |
|  |  |  |  | EKPFSCPH |
